# Supplementary material for: Genome-Wide CpG Island Methylation Profiles of Cutaneous Skin with and without HPV Infection
Source: Int J Mol Sci. 2019 Sep 28;20(19):4822. doi: 10.3390/ijms20194822 (PMC6801420; doi:10.3390/ijms20194822)
Supplement: Supplementary file 1 [file ijms-20-04822-s001.zip › Figure S1.docx]

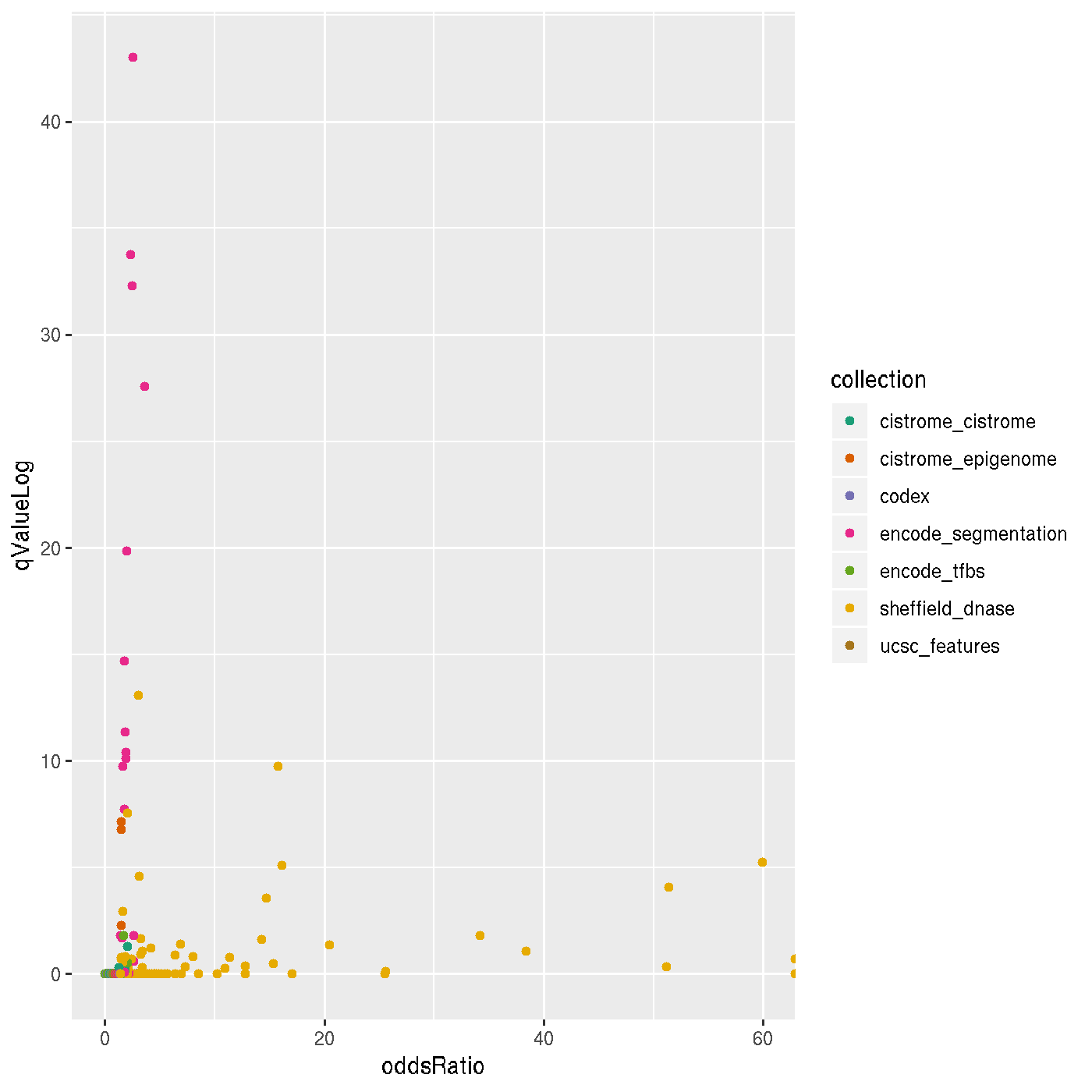


**Figure S1: Scatterplot of LOLA enrichment analysis showing the effect size (log(odds ratio)) vs the significance (-log10(q-value)) of the top 1000 hypermethylated CpG islands.** The LOLA reference database collections used are the cistrome database from Cistrome (cistrome_cistrome), epigenome database from Cistrome (cistrome_epigenome), codex database (codex), segmentation from ENCODE (encode_segmentation), transcription factor binding sites from ENCODE (encode_tfbs), tissue-clustered DNase hypersensitive sites from Sheffield (sheffield_dnase), and a collection of UCSC feature tables (ucsc_features).
