# Supplementary material for: Genome-Wide CpG Island Methylation Profiles of Cutaneous Skin with and without HPV Infection
Source: Int J Mol Sci. 2019 Sep 28;20(19):4822. doi: 10.3390/ijms20194822 (PMC6801420; doi:10.3390/ijms20194822)
Supplement: Supplementary file 1 [file ijms-20-04822-s001.zip › Figure S4.docx]

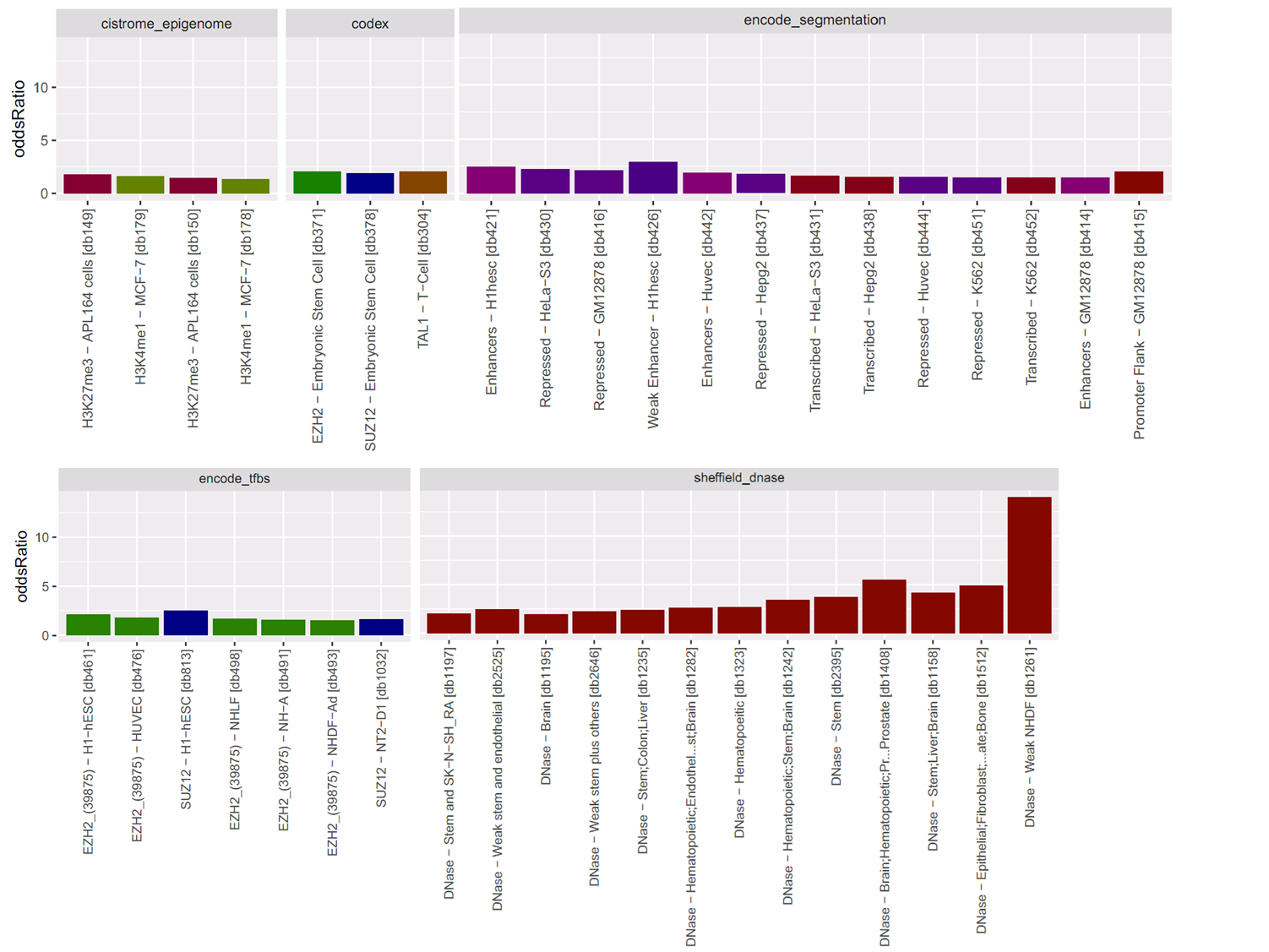


**Figure S4:** Bar plots of LOLA enrichment analysis showing the log(odds ratio) of the top 1000 hypomethylated CpG islands**.** Terms that exhibit statistical significance (p-value < 0.01) are shown. If more than 100 terms are enriched, the 100 terms receiving the highest joined LOLA ranks are shown. Coloring of the bars reflects the putative targets of the terms.
