# Supplementary material for: Genome-Wide CpG Island Methylation Profiles of Cutaneous Skin with and without HPV Infection
Source: Int J Mol Sci. 2019 Sep 28;20(19):4822. doi: 10.3390/ijms20194822 (PMC6801420; doi:10.3390/ijms20194822)
Supplement: Supplementary file 1 [file ijms-20-04822-s001.zip › Table S1.docx]

***Table S1.*** The 100 top-ranking CpG islands selected using the combined ranking score*.*

| **Gene** | **Chr** | **Start** | **End** | **Mean.mean β value (NS)** | **Mean.mean β value (W)** | **Mean.mean β value difference (W-NS)** | **Mean.mean.quot.log2** | **Comb.p.val** | **Comb.p.adj.fdr** | **Combined rank** |
| --- | --- | --- | --- | --- | --- | --- | --- | --- | --- | --- |
| ITGB5 | chr3 | 124860570 | 124861019 | 0.113 | 0.443 | 0.330 | 1.825 | 1.87E-12 | 9.91E-09 | 5 |
| … | chr19 | 1851579 | 1852203 | 0.192 | 0.465 | 0.273 | 1.280 | 3.53E-13 | 2.34E-09 | 13 |
| … | chr1 | 26097905 | 26098107 | 0.194 | 0.454 | 0.260 | 1.188 | 1.01E-11 | 3.34E-08 | 21 |
| … | chr9 | 100619985 | 100620192 | 0.194 | 0.541 | 0.347 | 1.433 | 3.72E-10 | 4.29E-07 | 23 |
| DTNB | chr2 | 25475707 | 25475928 | 0.276 | 0.530 | 0.253 | 1.118 | 1.16E-13 | 1.38E-09 | 27 |
| RBFOX3 | chr17 | 79377530 | 79377870 | 0.134 | 0.424 | 0.291 | 1.553 | 5.01E-10 | 4.75E-07 | 28 |
| SLC6A9, AL139220.2 | chr1 | 44031287 | 44031853 | 0.310 | 0.654 | 0.344 | 1.193 | 7.49E-10 | 6.41E-07 | 31 |
| C2orf27A | chr2 | 131720820 | 131721867 | 0.199 | 0.467 | 0.269 | 1.233 | 7.78E-10 | 6.46E-07 | 32 |
| MLLT1 | chr19 | 6271960 | 6272176 | 0.185 | 0.438 | 0.253 | 1.093 | 1.41E-11 | 4.16E-08 | 32 |
| ZNF407 | chr18 | 74691263 | 74691466 | 0.584 | 0.319 | -0.265 | -1.049 | 1.56E-13 | 1.38E-09 | 37 |
| PSMD2 | chr3 | 184301118 | 184302134 | 0.102 | 0.361 | 0.259 | 1.888 | 1.50E-09 | 1.05E-06 | 38 |
| LINC01150 | chr11 | 1903079 | 1903334 | 0.223 | 0.444 | 0.221 | 1.038 | 9.33E-10 | 7.50E-07 | 40 |
| TEX11 | chrX | 70823514 | 70824103 | 0.632 | 0.333 | -0.300 | -1.026 | 5.21E-11 | 1.09E-07 | 44 |
| ACR, AC000036.1 | chr22 | 50737810 | 50738961 | 0.117 | 0.323 | 0.205 | 1.249 | 2.58E-11 | 6.85E-08 | 48 |
| SLC39A11 | chr17 | 73073314 | 73073941 | 0.167 | 0.370 | 0.203 | 1.263 | 6.80E-09 | 3.68E-06 | 51 |
| CD37, TEAD2 | chr19 | 49340489 | 49340774 | 0.184 | 0.449 | 0.265 | 1.262 | 7.76E-09 | 4.04E-06 | 51 |
| … | chr4 | 169799087 | 169799625 | 0.363 | 0.732 | 0.369 | 0.993 | 1.45E-09 | 1.04E-06 | 54 |
| … | chr12 | 54811982 | 54812202 | 0.249 | 0.663 | 0.414 | 1.382 | 8.87E-09 | 4.36E-06 | 54 |
| SCARB1 | chr12 | 124873242 | 124874008 | 0.180 | 0.373 | 0.193 | 0.997 | 3.79E-12 | 1.68E-08 | 55 |
| … | chr6 | 50813315 | 50813699 | 0.306 | 0.611 | 0.305 | 0.975 | 4.77E-10 | 4.75E-07 | 57 |
| AL136018.1 | chr14 | 24641054 | 24642220 | 0.279 | 0.527 | 0.249 | 0.919 | 6.71E-09 | 3.68E-06 | 77 |
| CNOT1 | chr16 | 58535041 | 58535596 | 0.093 | 0.268 | 0.175 | 1.560 | 2.19E-08 | 8.32E-06 | 80 |
| … | chr10 | 105428506 | 105428713 | 0.239 | 0.448 | 0.209 | 0.871 | 5.35E-11 | 1.09E-07 | 95 |
| … | chr6 | 168684603 | 168684934 | 0.361 | 0.194 | -0.168 | -0.870 | 5.71E-08 | 1.55E-05 | 98 |
| CHMP1A | chr16 | 89646179 | 89647790 | 0.483 | 0.240 | -0.243 | -0.980 | 6.26E-08 | 1.59E-05 | 103 |
| NTRK1 | chr1 | 156863416 | 156863711 | 0.303 | 0.472 | 0.169 | 0.907 | 6.62E-08 | 1.64E-05 | 107 |
| HECW1 | chr7 | 43288635 | 43288948 | 0.430 | 0.238 | -0.193 | -0.839 | 4.22E-08 | 1.30E-05 | 110 |
| … | chr10 | 105452339 | 105453230 | 0.132 | 0.322 | 0.190 | 1.118 | 9.20E-08 | 2.09E-05 | 117 |
| JDP2 | chr14 | 75447512 | 75447779 | 0.318 | 0.559 | 0.241 | 0.826 | 5.68E-10 | 5.02E-07 | 118 |
| … | chr20 | 60905898 | 60906226 | 0.590 | 0.317 | -0.273 | -0.890 | 9.31E-08 | 2.09E-05 | 118 |
| CYP46A1 | chr14 | 99711840 | 99713431 | 0.151 | 0.414 | 0.263 | 1.516 | 1.05E-07 | 2.30E-05 | 121 |
| SMG5 | chr1 | 156261200 | 156261425 | 0.218 | 0.396 | 0.178 | 0.804 | 8.68E-09 | 4.35E-06 | 131 |
| … | chr1 | 2222199 | 2222569 | 0.291 | 0.456 | 0.165 | 0.790 | 1.07E-09 | 8.13E-07 | 139 |
| … | chr16 | 832828 | 833042 | 0.347 | 0.194 | -0.153 | -0.797 | 2.32E-08 | 8.66E-06 | 143 |
| … | chr10 | 105420686 | 105421076 | 0.361 | 0.623 | 0.262 | 0.784 | 4.66E-12 | 1.77E-08 | 145 |
| PNMA6A | chrX | 153072555 | 153072795 | 0.328 | 0.176 | -0.152 | -0.862 | 1.71E-07 | 3.24E-05 | 146 |
| DENND4A | chr15 | 65689143 | 65689362 | 0.377 | 0.220 | -0.158 | -0.771 | 2.05E-07 | 3.76E-05 | 149 |
| … | chr7 | 73720747 | 73720988 | 0.174 | 0.331 | 0.157 | 0.872 | 2.46E-07 | 4.26E-05 | 153 |
| PLEKHN1 | chr1 | 967967 | 970238 | 0.229 | 0.420 | 0.191 | 0.762 | 4.98E-08 | 1.44E-05 | 154 |
| MARK4, NKPD1 | chr19 | 45150372 | 45150834 | 0.355 | 0.605 | 0.250 | 0.762 | 5.13E-08 | 1.46E-05 | 155 |
| NLRX1 | chr11 | 119181397 | 119181670 | 0.394 | 0.247 | -0.147 | -0.942 | 1.35E-07 | 2.70E-05 | 162 |
| AC023509.3, ATF7, AC023509.6 | chr12 | 53591255 | 53591767 | 0.316 | 0.504 | 0.187 | 0.748 | 7.19E-08 | 1.72E-05 | 164 |
| … | chr16 | 85157831 | 85158048 | 0.474 | 0.273 | -0.201 | -0.740 | 3.84E-08 | 1.23E-05 | 170 |
| KRTAP5-AS1 | chr11 | 1592499 | 1592810 | 0.115 | 0.256 | 0.141 | 1.099 | 1.19E-07 | 2.51E-05 | 189 |
| PKP3 | chr11 | 394258 | 394619 | 0.466 | 0.279 | -0.188 | -0.751 | 5.69E-07 | 7.91E-05 | 191 |
| AMZ1 | chr7 | 2701331 | 2701744 | 0.372 | 0.210 | -0.162 | -0.789 | 6.84E-07 | 9.13E-05 | 199 |
| DGKZ | chr11 | 46366877 | 46367101 | 0.267 | 0.129 | -0.139 | -0.969 | 8.41E-09 | 4.29E-06 | 201 |
| PCNT | chr21 | 46378279 | 46378645 | 0.445 | 0.734 | 0.289 | 0.709 | 1.55E-08 | 6.55E-06 | 204 |
| … | chr18 | 77312680 | 77312970 | 0.486 | 0.288 | -0.198 | -0.743 | 8.01E-07 | 1.03E-04 | 207 |
| TRIP13 | chr5 | 912495 | 912861 | 0.263 | 0.126 | -0.138 | -0.998 | 4.32E-07 | 6.37E-05 | 212 |
| CHMP1A | chr16 | 89650793 | 89651058 | 0.450 | 0.245 | -0.205 | -0.847 | 9.21E-07 | 1.12E-04 | 218 |
| … | chr1 | 223936343 | 223937044 | 0.329 | 0.525 | 0.196 | 0.711 | 9.26E-07 | 1.12E-04 | 219 |
| IGHM | chr14 | 105851752 | 105851987 | 0.351 | 0.195 | -0.156 | -0.806 | 9.41E-07 | 1.12E-04 | 222 |
| CCDC40 | chr17 | 80058666 | 80058884 | 0.374 | 0.614 | 0.241 | 0.685 | 1.38E-10 | 2.44E-07 | 225 |
| … | chr10 | 5567123 | 5567828 | 0.283 | 0.148 | -0.135 | -0.890 | 3.15E-07 | 5.17E-05 | 227 |
| UGGT1 | chr2 | 128158438 | 128158912 | 0.354 | 0.183 | -0.171 | -0.944 | 1.04E-06 | 1.21E-04 | 228 |
| IFT140, AL133297.2 | chr16 | 1598953 | 1599157 | 0.444 | 0.266 | -0.178 | -0.733 | 1.05E-06 | 1.21E-04 | 230 |
| DAZAP1 | chr19 | 1424613 | 1424974 | 0.348 | 0.210 | -0.138 | -0.680 | 6.53E-07 | 8.84E-05 | 231 |
| SKI | chr1 | 2304160 | 2304376 | 0.439 | 0.300 | -0.139 | -0.674 | 4.17E-08 | 1.30E-05 | 236 |
| DTNB | chr2 | 25474758 | 25475598 | 0.069 | 0.203 | 0.134 | 1.466 | 3.45E-07 | 5.42E-05 | 239 |
| … | chr15 | 95388002 | 95388438 | 0.229 | 0.370 | 0.140 | 0.667 | 1.43E-07 | 2.81E-05 | 241 |
| AL591424.1 | chr9 | 138139508 | 138139727 | 0.284 | 0.152 | -0.133 | -0.863 | 5.22E-07 | 7.41E-05 | 244 |
| CBX8 | chr17 | 79799287 | 79799624 | 0.321 | 0.188 | -0.132 | -0.719 | 5.74E-07 | 7.93E-05 | 247 |
| CHAF1B | chr21 | 36398961 | 36399259 | 0.088 | 0.220 | 0.132 | 1.166 | 3.55E-07 | 5.54E-05 | 250 |
| GNB1 | chr1 | 1859247 | 1859523 | 0.397 | 0.230 | -0.166 | -0.802 | 1.37E-06 | 1.43E-04 | 253 |
| KAZN | chr1 | 13909607 | 13909842 | 0.312 | 0.476 | 0.165 | 0.658 | 1.29E-06 | 1.38E-04 | 257 |
| VGLL4 | chr3 | 11610138 | 11610370 | 0.435 | 0.185 | -0.250 | -1.138 | 1.68E-06 | 1.69E-04 | 265 |
| … | chr19 | 46270163 | 46270420 | 0.324 | 0.508 | 0.184 | 0.652 | 9.07E-07 | 1.12E-04 | 265 |
| MORN1 | chr1 | 2374819 | 2375814 | 0.108 | 0.237 | 0.129 | 0.952 | 1.73E-06 | 1.71E-04 | 270 |
| … | chr4 | 1772007 | 1772265 | 0.333 | 0.167 | -0.166 | -0.958 | 1.93E-06 | 1.87E-04 | 274 |
| AP003393.1 | chr11 | 119613006 | 119613521 | 0.084 | 0.212 | 0.128 | 1.162 | 3.23E-07 | 5.17E-05 | 274 |
| … | chr11 | 56947947 | 56948184 | 0.122 | 0.338 | 0.216 | 1.378 | 2.07E-06 | 1.97E-04 | 278 |
| KLF16 | chr19 | 1856726 | 1857443 | 0.075 | 0.202 | 0.127 | 0.942 | 8.53E-07 | 1.07E-04 | 283 |
| … | chr17 | 38347534 | 38347765 | 0.087 | 0.261 | 0.175 | 1.598 | 2.23E-06 | 2.08E-04 | 284 |
| … | chr22 | 38516736 | 38517030 | 0.314 | 0.187 | -0.127 | -0.715 | 1.06E-07 | 2.30E-05 | 284 |
| CCNL2 | chr1 | 1396086 | 1396338 | 0.449 | 0.299 | -0.150 | -0.636 | 2.50E-09 | 1.55E-06 | 293 |
| DLGAP2 | chr8 | 748281 | 748544 | 0.288 | 0.162 | -0.126 | -0.814 | 4.53E-09 | 2.61E-06 | 296 |
| MYPOP | chr19 | 45901453 | 45901688 | 0.448 | 0.685 | 0.237 | 0.635 | 1.90E-10 | 2.82E-07 | 297 |
| TFE3 | chrX | 49032262 | 49032567 | 0.281 | 0.145 | -0.136 | -0.892 | 2.61E-06 | 2.30E-04 | 301 |
| … | chr9 | 139715664 | 139716441 | 0.419 | 0.643 | 0.224 | 0.631 | 1.34E-08 | 6.11E-06 | 303 |
| HIF3A | chr19 | 46318491 | 46319266 | 0.406 | 0.620 | 0.215 | 0.626 | 1.91E-08 | 7.36E-06 | 309 |
| … | chr22 | 51016254 | 51017020 | 0.359 | 0.541 | 0.182 | 0.625 | 1.53E-06 | 1.56E-04 | 311 |
| … | chr9 | 140329455 | 140329710 | 0.274 | 0.129 | -0.145 | -1.028 | 3.27E-06 | 2.79E-04 | 312 |
| PTTG1IP | chr21 | 44863911 | 44864601 | 0.336 | 0.492 | 0.156 | 0.624 | 2.24E-07 | 3.99E-05 | 313 |
| EIF4G1 | chr3 | 184319377 | 184319822 | 0.324 | 0.166 | -0.158 | -0.922 | 3.43E-06 | 2.89E-04 | 315 |
| RNF216 | chr7 | 5647658 | 5647984 | 0.303 | 0.179 | -0.124 | -0.760 | 4.25E-07 | 6.30E-05 | 315 |
| KIF26A | chr14 | 104165052 | 104165278 | 0.331 | 0.208 | -0.123 | -0.649 | 1.96E-06 | 1.89E-04 | 317 |
| … | chr2 | 218843461 | 218843742 | 0.252 | 0.481 | 0.228 | 0.916 | 3.53E-06 | 2.95E-04 | 318 |
| MED16 | chr19 | 891532 | 892150 | 0.319 | 0.187 | -0.131 | -0.736 | 3.61E-06 | 2.99E-04 | 320 |
| CPXM2, AC068058.1 | chr10 | 123922851 | 123923542 | 0.351 | 0.540 | 0.190 | 0.620 | 2.25E-06 | 2.08E-04 | 324 |
| AC011933.4, GRB2 | chr17 | 75368689 | 75370506 | 0.101 | 0.222 | 0.122 | 1.116 | 5.37E-08 | 1.49E-05 | 331 |
| OPHN1 | chrX | 68060179 | 68060558 | 0.438 | 0.302 | -0.135 | -0.617 | 3.52E-08 | 1.15E-05 | 331 |
| AXIN1 | chr16 | 318618 | 318901 | 0.329 | 0.208 | -0.121 | -0.665 | 2.17E-06 | 2.05E-04 | 337 |
| … | chr1 | 113265574 | 113265787 | 0.122 | 0.243 | 0.121 | 0.958 | 1.22E-06 | 1.34E-04 | 338 |
| AP001360.2 | chr11 | 120008047 | 120008710 | 0.315 | 0.194 | -0.120 | -0.672 | 7.35E-08 | 1.74E-05 | 345 |
| … | chr18 | 21199432 | 21199798 | 0.036 | 0.161 | 0.126 | 1.885 | 5.13E-06 | 3.89E-04 | 350 |
| TTBK1 | chr6 | 43251672 | 43252018 | 0.424 | 0.280 | -0.144 | -0.606 | 5.06E-06 | 3.86E-04 | 351 |
| C2orf42 | chr2 | 70188512 | 70188726 | 0.670 | 0.443 | -0.227 | -0.631 | 5.38E-06 | 4.04E-04 | 353 |
| … | chr3 | 193851700 | 193852042 | 0.147 | 0.266 | 0.119 | 0.816 | 6.35E-08 | 1.59E-05 | 354 |
| … | chr20 | 33879905 | 33880215 | 0.388 | 0.253 | -0.135 | -0.604 | 7.44E-07 | 9.68E-05 | 358 |

Chr = chromosome; mean.mean β value = mean of mean methylation levels across all sites in a region; NS = normal skin; W = wart; mean.mean.quot.log2 = mean quotient in means across all sites in a region; comb.p.val = combined p-value; comb.p.adj.fdr = adjusted combined p-value.
